# The Role of Neutrophils in Corneal Wound Healing in HO-2 Null Mice

**DOI:** 10.1371/journal.pone.0021180

**Published:** 2011-06-17

**Authors:** Giuseppina Marrazzo, Lars Bellner, Adna Halilovic, Giovanni Li Volti, Filippo Drago, Michael W. Dunn, Michal Laniado Schwartzman

**Affiliations:** 1 Department of Pharmacology and Ophthalmology, New York Medical College, Valhalla, New York, United States of America; 2 Department of Drug Sciences Section of Biochemistry, University of Catania, Catania, Italy; 3 Department of Experimental and Clinical Pharmacology, University of Catania, Catania, Italy; Universidade de Sao Paulo, Brazil

## Abstract

Our studies demonstrated that Heme oxygenase (HO), in particular, the constitutive HO-2, is critical for a self-resolving inflammatory and repair response in the cornea. Epithelial injury in HO-2 null mice leads to impaired wound closure and chronic inflammation in the cornea. This study was undertaken to examine the possible relationship between HO-2 and the recruitment of neutrophils following a corneal surface injury in wild type (WT) and HO-2 knockout (HO-2^−/−^) mice treated with Gr-1 monoclonal antibody to deplete peripheral neutrophils. Epithelial injury was performed by removing the entire corneal epithelium. Infiltration of inflammatory cell into the cornea in response to injury was higher in HO-2^−/−^ than in WT. However, the rate of corneal wound closure following neutrophil depletion was markedly inhibited in both WT and HO-2^−/−^ mice by 60% and 85%, respectively. Neutropenia induced HO-1 expression in WT but not in HO-2^−/−^ mice. Moreover, endothelial cells lacking HO-2 expressed higher levels of the Midkine and VE-cadherin and displayed strong adhesion to neutrophils suggesting that perturbation in endothelial cell function caused by HO-2 depletion underlies the increased infiltration of neutrophils into the HO-2^−/−^ cornea. Moreover, the fact that neutropenia worsened epithelial healing of the injured cornea in both WT and HO-2^−/−^ mice suggest that cells other than neutrophils contribute to the exaggerated inflammation and impaired wound healing seen in the HO-2 null cornea.

## Introduction

In addition to being an important component of the refractive system of the eye, the cornea serves to protect the more delicate structures of the anterior segment of the eye from injury. It represents the initial barrier to the external environment and is in intimate and continuous contact with microorganisms and toxins, thus, constantly threatened by processes and agents which cause tissue injury and inflammation. Despite this challenge, this tissue is avascular, transparent and shows an extraordinary capacity for epithelial regeneration while maintaining a unique immune-privileged environment. Injury to the corneal surface elicits an inflammatory and repair response that, if is orderly executed, the structure and function of the cornea is restored. The corneal inflammatory response is marked by activation of corneal cells, in particular, stromal keratocytes, and recruitment of leukocytes to produce lipid and protein mediators that initiate, amplify and ultimately resolve the inflammation. The repair response, i.e., re-epithelialization process, proceeds simultaneously with migration and proliferation of epithelial cells. Aberrant activation of these pathways can lead to tissue destruction, ulceration, perforation and neovascularization, and ultimately to loss of vision [1], [2].

The recruitment of inflammatory cells into the injured cornea is a critical step in initiation and amplification of not only the inflammatory response (increasing innate immunity to prevent infection) but also the repair and healing process. Neutrophil migration into the corneal stroma following epithelial surface injury is evident within a few hours of injury. These neutrophils infiltrate the corneal stroma through the limbal blood vessels [1], [3], and their progression through the stroma to the area of the wound has been suggested to be facilitated by keratocyte apoptosis [4]. Neutrophil infiltration is an immediate response to injury, exerting their phagocytic functions to clear pathogens and cellular debris. However, at the same time neutrophils release into the injured tissue oxidative, hydrolytic and pore-forming molecules capable of damaging otherwise-healthy host cells. As such, an exaggerated neutrophil recruitment in response to injury or inflammatory stimuli contributes to the immunopathology observed in many diseases [5], [6]. The role of the neutrophil in the inflammatory response and healing of the cornea is controversial. Depletion of neutrophils or inactivation of their migration have been shown to both increase and decrease re-epithelialization and healing of the corneal surface [4], [7]–[9].

The heme oxygenase (HO) system has been implicated in the resolution of inflammation [10]. HO is the rate-limiting enzyme in heme catabolism. It cleaves heme to biliverdin, carbon monoxide (CO), and iron; biliverdin is subsequently converted by biliverdin reductase to bilirubin. Two isoforms, HO-1 and HO-2, are expressed in most tissues. HO-1 is an inducible enzyme, whereas HO-2 displays, in general, a constitutive expression that is developmentally regulated [11]. Our previous study showed that a deficiency in HO activity, as in the HO-2 null (HO-2^−/−^) mice, exacerbates ocular surface inflammation allowing an acute inflammation to become chronic with the stigma of chronic corneal inflammation such as neovascularization, ulceration, and perforation [12]–[14]. The present study was undertaken to examine the role that neutrophils play in the exaggerated inflammation developed in the HO-2^−/−^ mice. Our results demonstrated that the elevated number of neutrophils recruited to the injured tissues in the HO-2^−/−^ mice are not solely responsible for the delay in healing, on the contrary, the lack of neutrophils, through depletion, impairs significantly the wound healing in both wild type (WT) and HO-2 mice.

## Materials and Methods

### Animals

All animal experiments were performed following a protocol approved by the Institutional Animal Care & Use Committee (Approval # 40-2-0410.2) and in accordance with the National Institute's of Health Guide for the Care and Use of Laboratory Animals. The HO-2-null mice are direct descendants of the HO-2 mutants produced by Poss and colleagues [15]. These well-characterized HO-2-null mice have a C57BL/6x129/Sv genetic background [16], which was used on age- and gender- matched controls (Jackson Laboratory, Bar Harbor, ME). DNA was isolated from the tail and genotyped using HO-2 and Neo specific primers as described [16]. Neutropenia was induced with an intraperitoneal injection of 1.6 mg/Kg of Anti-Mouse Ly-6G (Gr-1), a monoclonal antibody (eBiosience, San Diego, CA) that specifically reacts with Ly-6G, which is present on neutrophils but not with Ly-6C, which is expressed on neutrophils, dendritic cells, and subpopulations of lymphocytes and monocytes [17], 24 hours prior to epithelial injury. Mice were anesthetized with ketamine (50 mg/kg) and xylazine (20 mg/kg) intramuscularly, and a drop of tetracaine-HCl 0.5% was applied to the eye to deliver local corneal anesthesia before animals were subjected to injury. The corneal epithelium was removed up to the corneal/limbal border with a 0.5-mm corneal rust ring remover (Algerbrush II; Alger Equipment, Lago Vista, TX). Wound closure (re-epithelialization) was monitored by fluorescein staining. Images of the anterior surface were taken with a dissecting microscope (Carl Zeiss, Jena, Germany) coupled to a digital camera (Axiocam HRc; Carl Zeiss) and analyzed using Zeiss software (Axiovision 4.5). Mice were sacrificed 2 days after injury, eyes were removed, and corneas free of conjunctival tissue were dissected and processed for selected analyses.

### Real-Time Polymerase Chain Reaction (PCR)

Total RNA was isolated using RNeasy Protect Mini Kit (QIAGEN, Carlsbad, CA) and RNA was quantified by Nano drop. Reverse transcription reaction of total RNA was performed using the qScript cDNA synthesis kit (Quanta Bioscience, Gaithersburg, MD). Quantitative real-time PCR was performed using PerfeCTa SYBR Green QPCR FastMix (Quanta Bioscience, Gaithersburg, MD) and the Mx3000 real-time PCR system (Stratagene, La Jolla, CA). Specific primers were designed based on published sequences (GenBank) and were as follows: HO-1 sense, 5′-TGCTCAACATCCAGCTCTTT-3′ and anti-sense, 5′-GCAGAATCTTGACTTTGTT-3′; HO-2 sense, 5′-ATGTCAGCGGAAGTGGAAAC-3′ and anti-sense, 5′-CGAGAGGTCAGCCATTCTCA-3′; Midkine (Mdk) sense, 5′-GAAGAAGGCGCGGTACAATG-3′ and anti-sense, 5′-GAGTGGATTCTGCATAATGG-3′; VE-cadherin sense, 5′-CGGCCACGCCACTGTCTTGT-3′ and anti-sense, 5′-CCAAGGGCTTGCCCACTCGG-3′; 18S sense, 5′-GATGGGCGGCGGAAAATAG-3′ and anti-sense, 5′-GCGTGGATTCTGCATAATGG-3′. PCR efficiency for each primer pair was determined by quantifying amplification with increasing concentrations of template cDNA, and specific amplification was verified by subsequent analysis of melt curve profiles for each amplification. A non-template control served as negative control to exclude the formation of primer dimers or any other nonspecific PCR products. RNA expression of target genes was calculated based on the real-time PCR efficiency (E) and the threshold crossing point (CP) and is expressed in comparison to the reference gene 18S as described [13].

### Isolation of Neutrophils

Neutrophils were isolated from peripheral blood. Briefly, blood was collected in EDTA-containing tubes. Most of the erythrocytes were removed by sedimentation using Anticoagulant Citrate Dextrose Solution (ACD) and dextran (6% dextran/PBS). The remaining erythrocytes and platelets were removed using ddH_2_O and 0.6 M KCl. Neutrophils were then isolated using Histopaque 1077 density centrifugation. Isolated neutrophils were re-suspended in Hank's Balanced Salt Solution. Isolation steps were performed at 4°C. The number of neutrophils purified were counted using hemacytometer (Fisher Scientific, Pittsburgh, PA). The yield was approximately 0.15–0.2×10^6^ neutrophils/ml blood.

### Histology and immunostaining

Dissected corneas were washed twice with PBS and embedded in OCT compound (Sakura Finetek, Torrence, CA). Croystat sections were cut transversely into 7 µm thick sections, stained with Hematoxylin-Eosin and mounted on microscopic slides in Cytosol XYL (Richard-Allan Scientific, Kalamazoo, MI). Immunofluorescence staining was performed using the following antibodies: rat anti-mouse Ly-6G (Gr-1) monoclonal antibody (1∶200, eBioscence, San Diego, CA), rat anti-mouse CD68 antibody (1∶100, AbD Serotec, Raleigh, NC). Briefly, frozen corneal sections were fixed in 4% paraformaldehyde- PBS for 15 min at room temperature and then blocked in 5% goat serum in PBS-Triton X100 (0.1%) for 1 h at room temperature. Then, sections were incubated with either rat anti-mouse Gr-1 or rat anti-mouse CD68 antibody (eBioscence, San Diego, CA) overnight at 4°C, washed and further incubated with a Cy3- conjugated goat anti-rat antibody (1∶500, Jackson Immunoresearch, West Grove, PA) for 2 h at room temperature. To further verify cellular entity, sections were washed and counterstained for nuclei with 4′,6- diamino-2-phenylindole (DAPI) for 15 minutes. Immunofluorescence was visualized using a Zeiss Axioplan-2 fluorescent microscope. Images were captured and analyzed using AxioVision 2 multi channel image processing software (Zeiss, Gottingum, Germany) [12].

### Adhesion assay

Isolation and culture of aortic endothelial cells (mAEC) from WT and HO-2^−/−^ mice were done as previously described [18]. Adhesion of neutrophils to the mAEC monolayer was measured using a CytoSelect Adhesion Assay Kit (Cell Biolabs, San Diego, CA) according to manufacturer's instructions. Endothelial cells were grown in a 48-well plate and treated with TNF-α (50 ng/ml) or its vehicle control for 8 h. Neutrophils were labeled with LeukoTracker, added to the wells, and incubated for 60 min at 37°C in a cell culture incubator. The mAEC were washed to remove the non-adherent neutrophils, lysed and fluorescence at 480 nm/520 nm was measured with a Synergy HT Multi-Mode Microplate Reader (Biotek, Winooski, VT). Neutrophil adhesion was expressed as relative fluorescence units (RFU).

### Statistical Analysis

Student's t-test was used to evaluate the significance of differences between groups, and multiple comparisons were performed by regression analysis and one-way analysis of variance. P<0.05 was considered significant. All data are presented as mean ± SE.

## Results

### Neutrophil depletion impairs wound healing in both WT and HO-2^−/−^ mice

**Figure 1:**
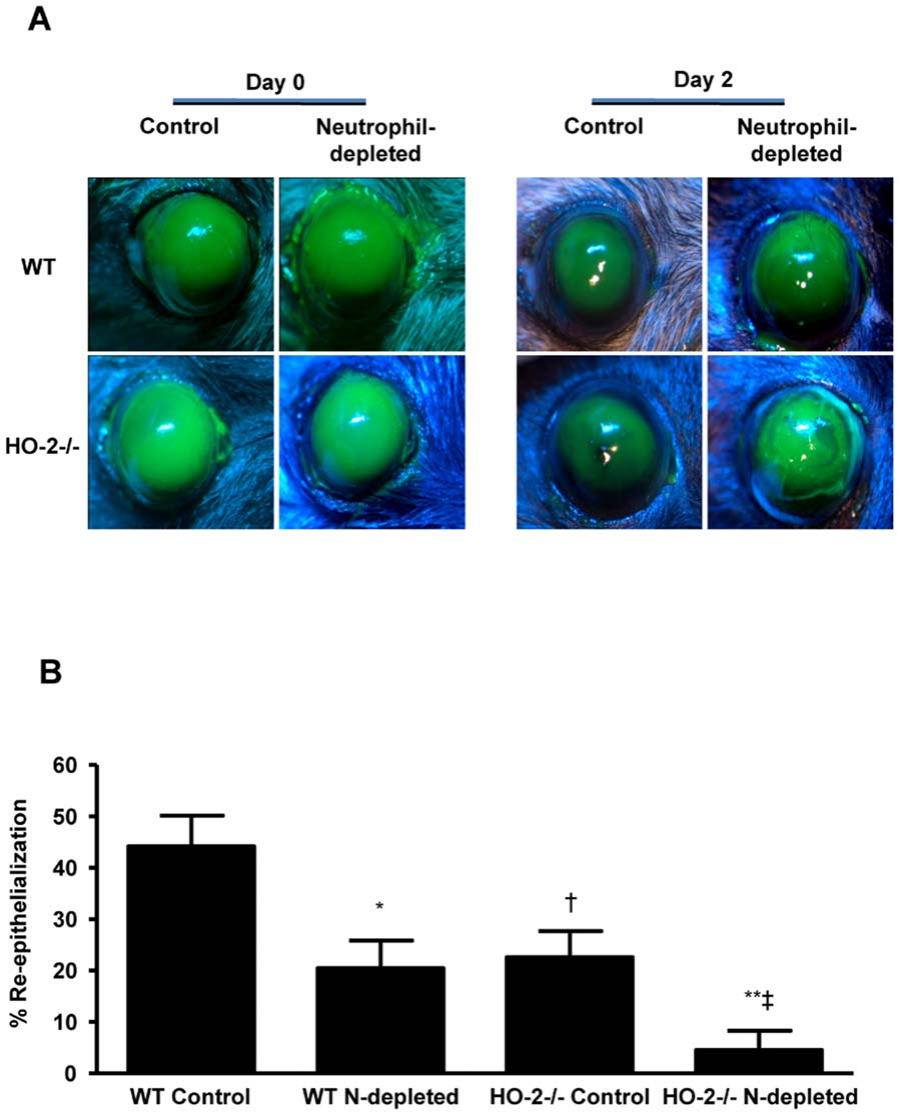
Effect of neutrophil depletion on wound healing. (**A**) Representative images of fluorescein-stained corneas at day 0 and day 2 after injury in control and neutrophil (N)-depleted WT and HO-2^−/−^ mice. (**B**) Wound closure as percent change from day 0. Results are mean ± SE; n = 5–7; *p<0.005 from control WT mice; **p<0.005 from control HO-2^−/−^ mice; † p<0.01 from control WT mice; ‡ p<0.005 from neutrophil-depleted WT mice.

The corneal epithelial injury model is a well-established model in which the inflammatory and reparative response has been well characterized [13], [19]. Wound healing was assessed in control and neutrophil-depleted WT and HO-2^−/−^ mice. As seen in Figure 1, epithelial injury in WT mice produced a consistent wound in control mice (6.6±0.3 mm^2^, n = 6) that exhibited 44.2%±5.2% re-epithelialization by day 2 after injury. In contrast, wound closure in neutrophil-depleted WT mice (7.2±0.2 mm^2^, n = 6) was markedly inhibited displaying re-epithelialization of 20.5%±4.0% at day 2 after injury. Similar results were obtained in the HO-2^−/−^ mice in which wound closure was further impaired following neutrophil depletion. Thus, epithelial injury in HO-2^−/−^ mice produced a consistent wound in control (7.4±0.1 mm^2^, n = 5) and neutrophil-depleted mice (7.6±0.2 mm^2^, n = 5) that was closed by 22.6%±4.9% and 4.5%±2.6%, respectively, at day 2 after injury (Figure 1 A and B).

### Neutrophil depletion reduces the number of infiltrated cells into the injured cornea

**Figure 2:**
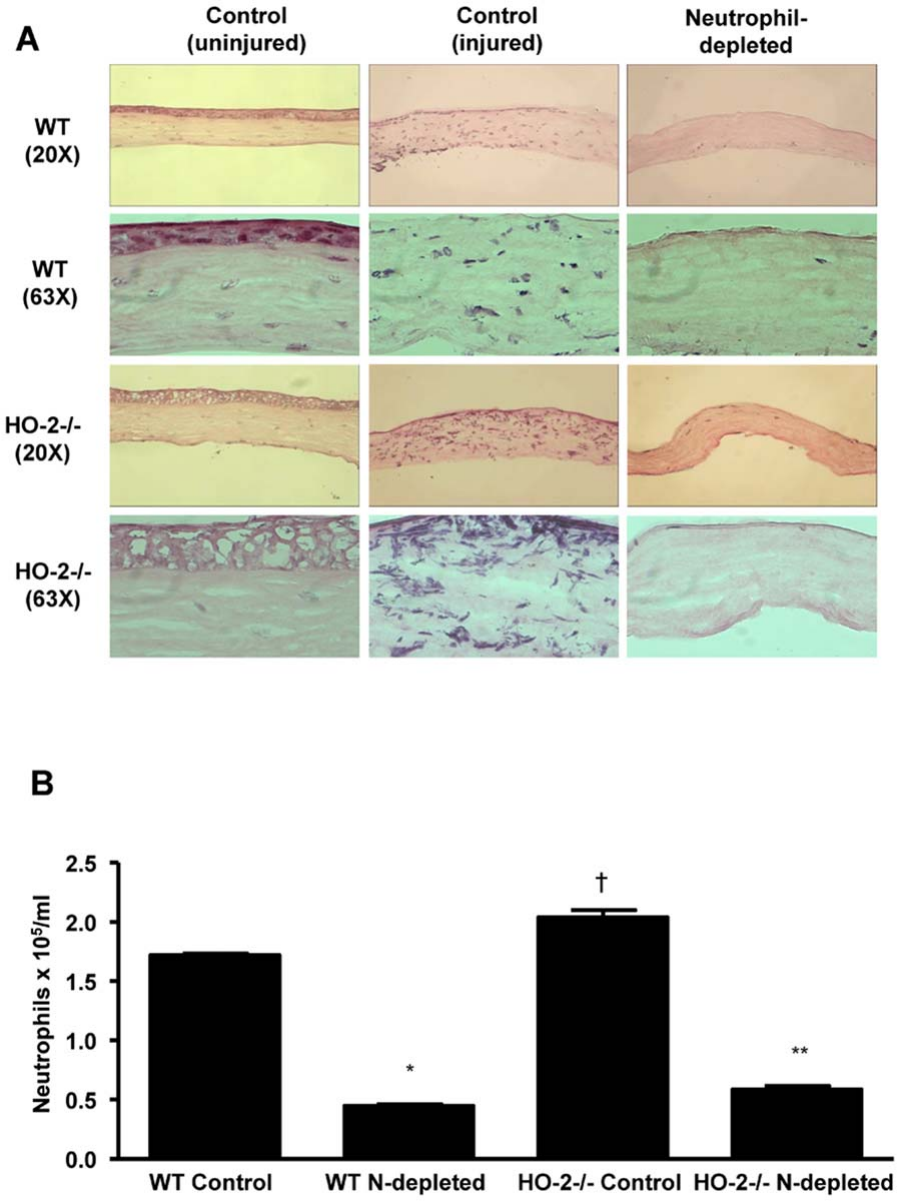
Infiltration of Inflammatory cells in the cornea at day 2 after epithelial injury. (**A**) Representative H&E staining of corneas from uninjured and injured control and neutrophil-depleted WT and HO-2^−/−^ mice 2 days after injury. (**B**) Number of neutrophils in peripheral blood of control and neutrophil (N)-depleted WT and HO-2−/− mice 2 days after injury. Results are mean ± SE; n = 3; *p<0.0001 from control WT; **p<0.0001 from control HO-2^−/−^ mice; † p<0.05 from control WT.

**Figure 3:**
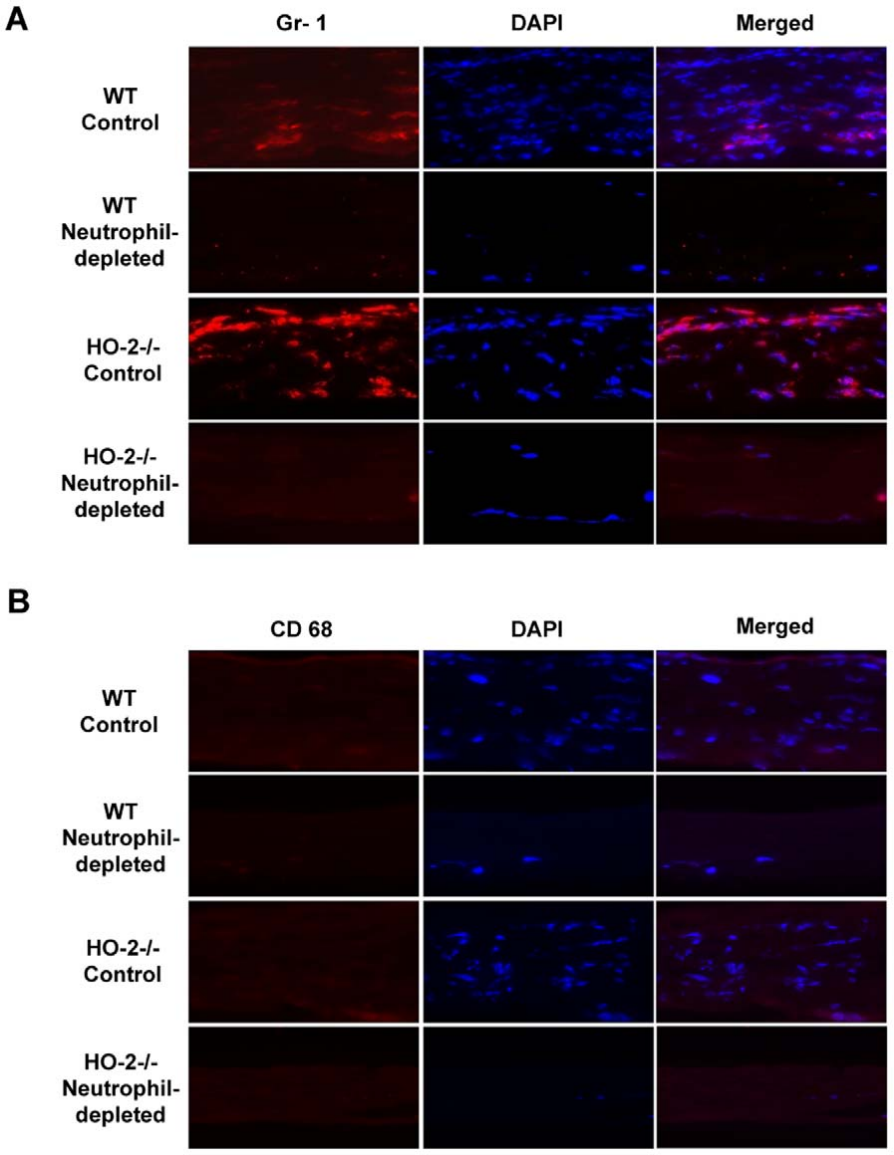
Immunofluoescence analysis of (A) Gr-1- and (B) CD68-positive cells in corneas from control and neutrophil-depleted WT and HO-2^−/−^ mice 2 days after injury. Images were taken at 63× magnification and are representative of three separate analyses.

H&E staining of corneal sections from neutrophil-depleted WT or HO-2^−/−^ clearly showed the absence of neutrophils (Figure 2A). The number of neutrophils harvested from control HO-2^−/−^ mice (not injected with Gr-1 antibody) was significantly higher than that in the corresponding WT mice (Figure 2B). However, injection of Gr-1 antibody to either WT of HO-2^−/−^ mice resulted in a comparable reduction of approximately 70% in the number of systemic neutrophils (Figure 2B). It should be noted that the number of neutrophils harvested from healthy uninjured or injured WT mice was similar and amounted to 1.68±0.03×10^5^/ml. The number of neutrophils harvested from healthy uninjured or injured HO-2−/− mice was similar and amounted to 1.98±0.07×10^5^/ml. The H&E staining also confirmed previous studies [12], [13] showing exaggerated neutrophil infiltration in response to epithelial injury in the HO-2^−/−^ cornea as compared to the WT corneas (Figure 2A). To further substantiate the nature of the cellular infiltrate, immunofluorescence analysis using the anti-mouse Ly-6G (Gr-1) antibody was performed. This antibody preparation is highly specific for neutrophils and have been used extensively to examine the role of neutrophils in innate immunity [17]. As seen in Figure 3A and B, Gr-1 positive staining was seen throughout the cornea; it was greater in the HO-2^−/−^ injured corneas as compared to WT corneas and was nearly absent in corneas of neutrophil-depleted mice of both genotypes. Although, within the time frame of the experiment (2 days post injury), neutrophils clearly constitute the majority (>90%) of the cellular infiltrate [4], [20], additional immunofluorescence staining for CD68 (a macrophage-specific marker)-positive cells showed little if any positive signals in the 2-day post injury corneas of WT or HO-2 null mice (Figure 3B).

### HO-1 expression in neutrophils

**Figure 4:**
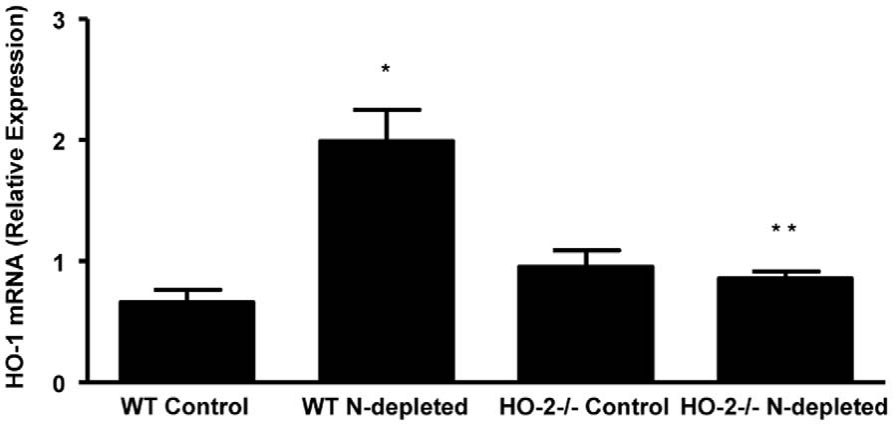
Real-time PCR analysis of HO-1 mRNA expression in neutrophils purified from peripheral blood of control and neutrophil (N)-depleted WT and HO-2^−/−^ mice 2 days after injury. Results are the mean ± SE; n = 3; *p<0.01 from neutrophils purified from control WT mice; **p<0.05 from neutrophils purified from neutrophil-depleted HO-2^−/−^ mice.

Neutrophils exhibited expression of both HO-1 and HO-2; however, HO-2 expression was absent in neutrophils from HO-2−/− (data not shown). The residual neutrophils in the systemic circulation after injection of Gr-1 antibody displayed a 3-fold higher expression of HO-1 mRNA level in neutrophils purified from WT-depleted mice as compared to neutrophils from control WT mice (Figure 4). Interestingly, while neutrophils from control HO-2−/− mice display similar HO-1 expression, the HO-1 expression failed to increase in the residual neutrophils from neutrophil-depleted HO-2−/− mice (Figure 4) in agreement with a previous study showing that HO-1 induction in the cornea as well as in stimulated peritoneal neutrophils is impaired in HO-2−/− mice [13].

### Neutrophil adhesion to HO-2 null endothelial cells

**Figure 5:**
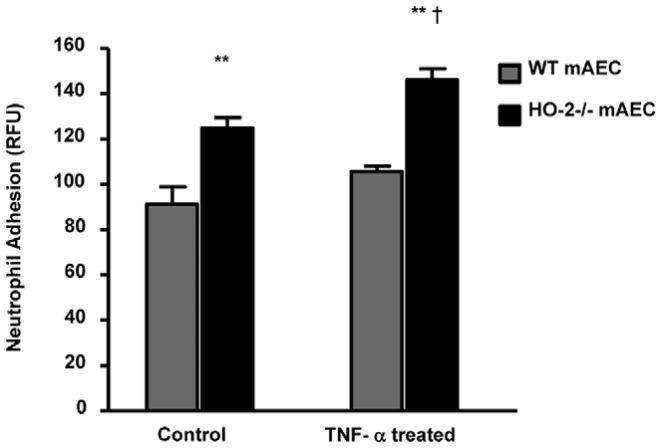
Neutrophil adhesion onto untreated (Control) and TNFα-treated WT and HO-2^−/−^ aortic endothelial cells (mAEC). Neutrophil adherence is measured as the fluorescence intensity associated with mAEC and is given in relative fluorescence units (RFU). Results are the mean ± SE; n = 3; **p<0.005 from WT mAEC; †p<0.05 from untreated HO-2^−/−^ mAEC.

**Figure 6:**
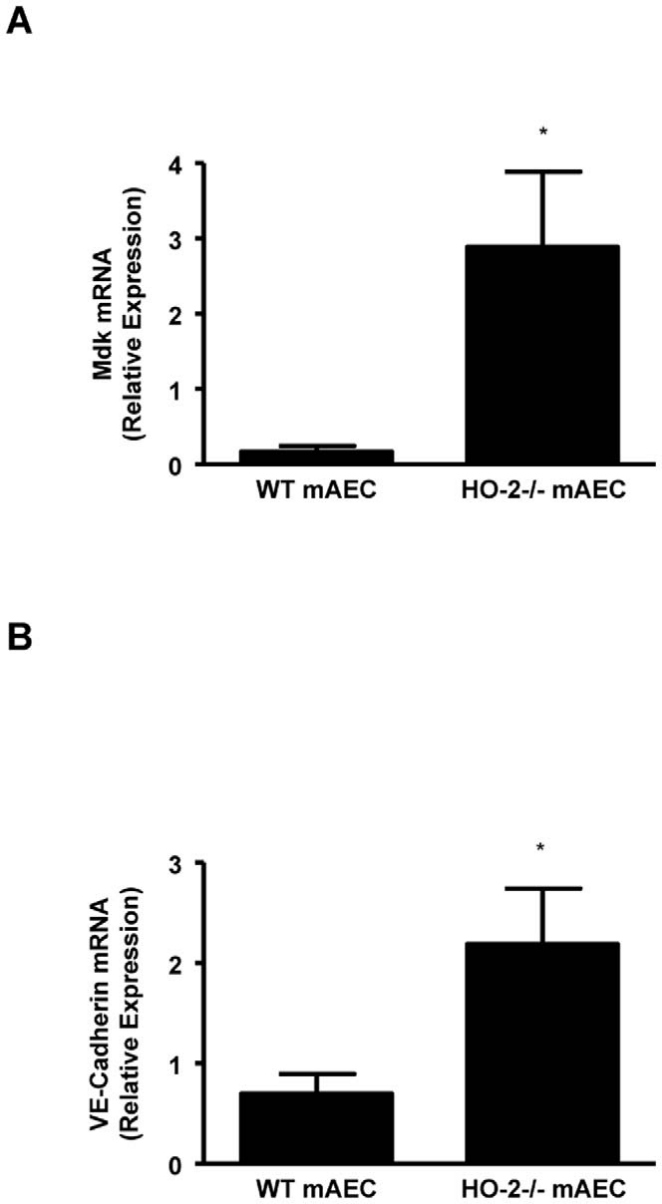
Real-time PCR analysis of mRNA expression of (A) midkine (Mdk) and (B) VE-Cadherin in untreated aortic endothelial cells (mAEC) from WT and HO-2^−/−^. Results are the mean ± SE; n = 4 to 5; *p<0.005 from WT mAEC.

Migration of neutrophils through the blood vessel wall is highly regulated by endothelial cells adhesion molecules. The role of HO-2 in neutrophil adhesion was examined using aortic endothelial cells lacking the HO-2 gene [18]. As seen in Figure 5, neutrophils adhere significantly more to HO-2−/− endothelial cells compared to WT mAEC. Moreover, this adhesion was enhanced in the in mAEC treated with TNF-α. Interestingly, mAEC from HO-2−/− mice showed a marked increase in the expression of Mdk, a 13-kDa multifunctional heparin-binding protein that promotes migration of neutrophils, macrophages, and neurons [21] (Figure 6A). The HO-2−/− mAEC also expressed significantly higher levels of VE-cadherin (Figure 6B), a key junctional molecule for transendothelial migration of neutrophils [22].

When examining adherence of neutrophils from HO-2^−/−^ mice to either endothelial cells from WT or HO-2^−/−^ mice, we found no significant difference. Thus, neutrophil adhesion in relative fluorescent units (RFU) to WT and HO-2^−/−^ endothelial cells amounted to 157±7 and 137±11, respectively (n = 6; p = 0.14). The results are interesting and raise the possibility that the interactions between neutrophils and endothelial cells in the HO-2^−/−^ mice are compromised by mechanisms that need to be further explored.

## Discussion

This study is a follow-up on previous studies that revealed a functional relationship between expression of HO-2 and neutrophil recruitment after corneal injuries. HO-2 null mice experience an exaggerated inflammatory response after injury marked by a 4-fold increase in neutrophil infiltration in the corneal stroma, which might be fueling the exaggerated inflammation seen in these mice [12], [13]. Infiltration of inflammatory cells into injured tissue is the hallmark of wound repair. Polymorphonuclear leukocytes, primarily neutrophils, are the first to migrate into the tissue in response to injury. Numerous studies in different types of ocular surface injury including epithelial abrasion, chemical burns and pathogenic insults have documented the presence of neutrophils within the corneal stroma as early as 6 h after injury [4], [8], [13], [19], [23]. These neutrophils transmigrate from the limbal vessels and through the stroma, presumably with the help of keratocytes, into the injured area [24]. The number of neutrophils in the injured cornea peaks 24–48 h after injury and begins decreasing, thereafter, as other cell types, such as macrophages begin to appear. Macrophages act in concert with neutrophils to phagocytose debris and invading pathogenic microorganisms and are a source of chemoattractants and growth factors that promote resolution of inflammation as well as cell migration and proliferation for wound closure and healing [10]. While infiltration of inflammatory cells is crucial for repair, their continuous influx and presence may be detrimental.

Our prior studies have determined that HO-2 is crucial for an ordered execution and, most importantly, resolution of acute inflammation that is essential for wound healing; in the absence of HO-2 activity, an acute inflammation becomes chronic with a continuous influx of inflammatory cells leading to tissue destruction. We have defined a cytoprotective and anti-inflammatory role for HO in the cornea in a series of publications [12], [13], [25]–[27]. This HO function is seen clearly during wound healing after an epithelial injury. Using HO-2 null mice that also express little HO-1, thereby, abrogating all HO activity, a normally self-resolving epithelial injury becomes a massive non-resolving inflammatory defect with attenuated wound healing, ulceration and perforation. These corneas can be rescued by topical application of biliverdin/bilirubin or carbon monoxide CO, both products of HO metabolism of heme [12]–[14]. Biliverdin/bilirubin and CO serve as stop signals controlling leukocyte migration and activation by attenuating adhesion molecule expression and cytokine and chemokine induction [12], [28]–[32]. Studies using models of epithelial injury [33]–[35] demonstrated spatial and temporal increases in HO-1 expression that correlated with leukocyte infiltration, keratinocyte proliferation, resolution and wound healing. In fact, HO-1 induction has been implicated as a switch for resolution [10]. The finding in this study together with previous studies demonstrating impaired HO-1 inducibility in the HO-2^−/−^ injured corneas and stimulated neutrophils [13] suggests that such impairment may contribute to increase neutrophil migration and activation, in sum, increasing tissue damage. The increased expression of Mdk, VE-cadherin and increased adherence of neutrophils to HO-2−/− mAEC cells is consistent with increased neutrophil migration and activation.

It is well known that a healthy corneal wound healing requires the timely recruitment of neutrophils. Absence of neutrophil recruitment impairs healing of the cornea, while exaggerated neutrophil infiltration coupled with impaired resolution leads to delayed and aberrant wound healing [7], [13]. Smith and colleagues [4], [24], using a model similar to ours, found that the main adhesion molecules involved in neutrophil activity during corneal inflammation are the CD18 integrin and the P and E-selectins. Re-epithelialization was significantly delayed in mice with deletion of these adhesion molecules and in mice made neutropenic. These results are consistent with ours: Neutrophil-depleted WT mice had a substantial delay in wound closure, as did the HO-2 null mice. The most profound delay was seen in HO-2 null mice that were also neutrophil-depleted. On the other hand, Ueno et al. [7], found accelerated wound closure in neutrophil-depleted mice. Their injury model was an alkali burn, which is a different injury from epithelial removal. Histological examination of our WT neutrophil-depleted corneas showed a complete absence of neutrophils as well as substantially delayed wound closure as compared to control WT corneas, which led us to conclude that neutrophil infiltration of the corneal stroma promotes epithelial, wound closure. Neutrophil-depleted WT mice corneas are also denied the benefit of infiltrating neutrophil-derived HO-1 and HO-2. This is further exaggerated in the HO-2−/− mice in which HO-1 inducibility is impaired [13]. In all, this suggests that HO-2 deficiency in corneal cells rather than in inflammatory cells contributes to the exaggerated inflammation and impaired wound healing seen in the HO-2^−/−^ cornea. It also suggests that perturbation in endothelial cell function caused by HO-2 deletion underlies the increased infiltration of neutrophils into the HO-2^−/−^ cornea. Undoubtedly, additional experiments are needed to distinguish the role of neutrophils and corneal cells in this model.

Interestingly, we noticed from our current study and previous reports that in WT the majority of neutrophils were present adjacent to the leading edge of the wound whereas in the HO-2−/− neutrophils are randomly distributed all through the stroma. This raises the possibility that HO-2 deletion impairs guidance of neutrophils to the injured area. It has been suggested that keratocyte network plays a prominent role in neutrophil migration by serving as a source of contact guidance and chemoattraction for migrating neutrophils [24], [36], [37]. Likewise, injured epithelial cells release signals that attract neutrophils [1]. The fact that neutropenia worsened epithelial healing of the injured cornea in both WT and HO-2 null mice suggest that cells other than neutrophils contribute to the exaggerated inflammation and impaired wound healing seen in the HO-2 null cornea [12]–[14]. Certainly, the role of HO-2 in epithelial and keratocyte cell function need to be further evaluated.

Endothelial cells lining the blood vessel wall are active participants in the inflammatory response and highly regulated endothelial-leukocyte interactions are in place so as to facilitate leukocyte entrance into the injured tissue [38]. Neutrophils infiltrating the injured tissue are derived from circulating leukocytes in the adjacent limbal vessels that have become transiently leaky in response to signals released by injured cells. Endothelial cell activation initiated at the time of injury is exemplified by increased release of cytokines and chemokines and by increased expression of adhesion molecules including P and E selectins, integrins and ICAM. Activated endothelial cells are “sticky” allowing circulating neutrophils to anchor and roll and ultimately enter the site of inflammation. In a recent study, we showed that vascular endothelial cells derived from HO-2−/− mice are highly activated; they display increased expression of cytokines and adhesion molecules [18]. Further analysis in the current study revealed that expression of the chemokine MDK and the adhesion molecule VE-Cadherin are greatly increased in HO-2−/− mAEC, both have been shown to regulate leukocyte-endothelial interactions. Different studies indicate that during an inflammatory process, its expression is increased; the response to the inflammatory stimuli was less severe in midkine knockout (Mdk−/−) mice than in WT mice and the numbers of infiltrating neutrophils and also macrophages were lower in Mdk−/− than in WT mice [39]. VE-Cadherin is a tight-junction protein specific to endothelial cells that is involved in the regulation of endothelium permeability and consequently to leukocyte-endothelial interactions [22], [40], [41]. It is possible that perturbation in endothelial cell function caused by HO-2 deletion underlies the increased infiltration of neutrophils into the HO-2−/− cornea.

In summary, the HO-2 null mice showed an exaggerated corneal inflammation associated with an increased number of inflammatory cells compared to WT. However, systemic and corneal neutrophil depletion worsened rather than improved the wound healing process in both WT and HO-2−/− mice, suggesting that the absence of HO-2 gene within corneal cells contributed to the impaired corneal healing in the HO-2 null mice. Increasing HO activity may be beneficial in the treatment of corneal inflammatory conditions such as the dry eye syndrome and in the treatment of non-healing corneal defects.
